# 2024: The quest continues

**DOI:** 10.1098/rsob.240010

**Published:** 2024-01-31

**Authors:** Jonathon Pines

**Affiliations:** Department of Cancer Biology, Institute of Cancer Research, London, UK

Two years ago, *Open Biology* was 10 years old and my editorial described our quest to make ‘publishing your research less frustrating and more constructive’. This year we have taken another step towards this by joining Review Commons (https://royalsociety.org/blog/2023/12/open%20biology%20joins%20review%20commons/). This is an exciting initiative from EMBO and ASAPbio that promises to reduce the time and effort to publish your work. As an author, you submit a preprint of your work to Review Commons for peer review. Once the reviews are completed, you can submit your preprint plus reviews plus your responses to *Open Biology* and, if it of interest to us, we agree to publish the revised manuscript. This should greatly reduce the time and emotional effort in publishing your work.

Our enthusiastic participation in Review Commons joins our other efforts to improve the publishing and peer review process, such as rewarding our reviewers with tokens that can be put towards the access charges for publishing, encouraging submissions from preprint servers and our ‘Opening Up’ (https://royalsocietypublishing.org/doi/10.1098/rsob.200334) section in research articles where you can put your papers in wider context and ruminate on the wider implications of your work. You can continue to ruminate if your paper is selected for our new ‘Cell and molecular biology’ (https://cassyni.com/publish/cellular-and-molecular-bio) seminar series in partnership with Cassyni. You will be invited to record a seminar about your paper, which will give you the opportunity both to expand on the context and implications of your work, and tell the often fascinating story of how it all came about. Alternatively, if you have a startling new observation that you want to publicise quickly, you can take advantage of our Short Communication format (https://royalsociety.org/blog/2023/07/open-access-discount-on-short-communications/) (and the reduced article processing charge until the end of this month!).

Since my last editorial, we have published our Special Issue on Quantitative imaging (https://royalsociety.org/blog/2023/12/open-biology-decoding-lifes-inner-workings/) guest edited by Ricardo Henriques, Christophe Leterrier and Aubrey Weigel that showcased the power of new forms of microscopy and image analysis to answer questions across the breadth of biological scales, ranging from super-resolution microscopy to map molecular complexes through probes to interrogate membrane properties [1], and from image analysis scripts to classify changes in the morphology of microglia [2], to the macroscale measurement of tissue mechanics in axolotl development [3]. This year we will address the scale of biological time and publish a Special Issue on Evolutionary Cell Biology of Microbial Eukaryotes, guest edited by Filip Husnik and Guatam Dey.

I would like to finish by thanking all who are so crucial to the success of *Open Biology*:

Our Subject Editors, with a special mention to our associate editors who finished their terms: Corrine, Houart, Claude Desplan, Lynne Reagan, Nicholas Tonks, Celia Shiau, Jean-Ju Chung, Jing-Wei Xiong, Anne Villeneuve and associate editors Izzy Jayasinghe, Yudan Ren, Gregorio Manuel Iraola Bentancor, Laura Andreae, Mayssa Mokalled, Kazuyo Moro, Anca Flavia Savulescu and Sandra Lopez-Verges who are joining us. We extend our thanks to Faith Osier, who is stepping down as our Immunology Subject Editor; our peer reviewers, who help to improve the science we publish; to the tireless editorial staff at *Open Biology*, notably Buchi Okereafor, who keep the wheels turning; and most of all to you, our authors and readers!
